# The two isoforms of matrix metalloproteinase- 2 have distinct renal spatial and temporal distributions in murine models of types 1 and 2 diabetes mellitus

**DOI:** 10.1186/s12882-018-1029-8

**Published:** 2018-09-25

**Authors:** Il Young Kim, Sang Soo Kim, Hye Won Lee, Sun Sik Bae, Hong Koo Ha, Eun Soon Jung, Min Young Lee, Miyeun Han, Harin Rhee, Eun Young Seong, Dong Won Lee, Soo Bong Lee, David H. Lovett, Sang Heon Song

**Affiliations:** 1 Biomedical Research Institute and Department of Internal Medicine, Pusan National University Hospital, Gudeok-ro 179 Seo-gu, Busan, 49241 Republic of Korea; 2 Research Institute for Convergence of Biomedical Science and Technology and Department of Internal Medicine, Pusan National University Yangsan Hospital, Yangsan, Gyeongsangnamdo Republic of Korea; 3 MRC for Ischemic Tissue Regeneration, Medical Research Institute, and Department of Pharmacology, Pusan National University School of Medicine, Yangsan, Republic of Korea; 4 Biomedical Research Institute and Department of Urology, Pusan National University Hospital, Busan, Republic of Korea; 5 The Department of Medicine, San Francisco Department of Veterans Affairs Medical Center, University of California San Francisco, California, USA

**Keywords:** Diabetes mellitus, Hyperglycemia, Matrix metalloproteinase-2, Oxidative stress

## Abstract

**Background:**

We recently reported on the enhanced tubular expression of two discrete isoforms of the MMP-2 (full length and N-terminal truncated, FL-MMP-2, NTT-MMP-2) in a murine model and human diabetic kidneys. In the present study, we examined in more detail the temporal and spatial distributions of MMP-2 isoform expression in murine models of Type 1 and Type 2 diabetes mellitus.

**Methods:**

Diabetic models were streptozotocin (STZ)-induced diabetes (Type 1 diabetes mellitus) and db/db mice (Type 2 diabetes mellitus). We quantified the abundance of two isoforms of MMP-2 transcripts by qPCR. A spatial distribution of two isoforms of MMP-2 was analyzed semi-quantitatively according to time after injection of STZ and with increasing age of db/db mice. Furthermore, immunohistochemistry for nitrotyrosine was performed to examine a potential association between oxidative stress and MMP-2 isoform expression.

**Results:**

Both isoforms of MMP-2 were upregulated in whole kidneys from STZ and db/db mice. In the case of FL-MMP-2, mRNA levels significantly increased at 12 and 24 weeks in STZ mice, while the isoform expression was significantly increased only at 16 weeks, in the db/db mice. FL-MMP-2 protein levels increased in the cortices and outer medullae of both STZ and db/db mice as a function of the duration of diabetes. For NTT-MMP-2, mRNA levels increased earlier at 4 weeks in STZ mice and at 10 weeks of age in db/db mice. The expression of NTT-MMP-2 also increased, primarily in the cortices of STZ and db/db mice, as a function of the duration of diabetes. Quantitatively, these findings were consistent with the qPCR results in the case of NTT-MMP-2, respectively (STZ 24 weeks, 3.24 ± 3.70 fold; 16 weeks db/db, 4.49 ± 0.55 fold). In addition, nitrotyrosine was expressed primarily in cortex as compared to medulla as a function of the duration of diabetes similar to NTT-MMP-2 expression.

**Conclusions:**

Two isoforms of MMP-2 are highly inducible in two diabetic murine models and become more abundant as a function of time. As the expression patterns were not the same in the two isoforms of MMP-2, it is possible that each isoform has a discrete role in the development of diabetic renal injury.

## Background

Diabetes mellitus (DM) is the most common lifestyle-related disease and a heavy public health burden. In addition, the prevalence of DM is increasing very rapidly worldwide [1]. More than 2.7 million Koreans aged 30 years or older have Type 2 DM and the prevalence of an elevated fasting glucose was approximately 25.0% in 2013 [2]. Moreover, diabetes mellitus is the most common etiology of end-stage renal disease (ESRD) and accounts for 48.4% of new ESRD patients in 2015 Korean Registry [3]. Diabetic nephropathy is the most representative renal complication and related to injury to renal tubular cells and various cells of glomeruli. Given the prevalence and the extent of progression to ESRD, further elucidation of core pathophysiologic mechanisms of diabetic nephropathy is an important research goal.

Our laboratories have identified matrix metalloproteinase-2 (MMP-2) as a central mediator of acute and chronic renal injury in both experimental and human clinical settings [4–7]. Transgenic renal tubular epithelial cell expression of the full length, secreted isoform of MMP-2 (FL-MMP-2) results in loss of tubular basement membrane integrity, with resultant tubular atrophy, interstitial fibrosis and inflammation [6]. More recently, we have identified a novel intracellular isoform of MMP-2, called N-terminal truncated isoform of MMP-2 (NTT-MMP-2) [8]. The NTT-MMP-2 isoform lacks a secretory sequence and the inhibitory propeptide and remains intracellular in association with mitochondria in an enzymatically active form [7, 8]. NTT-MMP-2 isoform activation contributed to tubular epithelial cell regulated necrosis, which induced by the mitochondrial permeability transition and enhancement of sensitivity to ischemia/reperfusion injury [5]. Moreover, we tried to explore the expression of two isoforms of MMP-2 in diabetic kidney and firstly reported on the enhanced tubular epithelial cell expression of the FL-MMP-2 and NTT-MMP-2 isoforms in the murine streptozotocin model of Type 1 diabetes mellitus and in archival renal biopsies from patients with diabetic nephropathy [9]. So, we hypothesized that two isoforms of MMP2 can be important mediators in diabetic injury and the current study was designed to expand on these initial observations and to determine if differences exist between the spatial and temporal patterns of MMP-2 isoform expression in murine models of Type 1 (streptozotocin) and Type 2 (db/db mice) diabetic nephropathy. The findings of this study will provide a solid foundation for future studies to uncover mechanisms related to diabetic renal injury.

## Methods

### Murine models of diabetic nephropathy

The animal protocol (2014–069, 2016–102) used in this study was reviewed and approved by the Pusan National University–Institutional Animal Care and Use Committee (PNU-IACUC) regarding their ethical procedures and scientific care. Mice were randomized into control and diabetic groups. The first diabetic murine model was induced by five daily intraperitoneal injections of streptozotocin (STZ, 40 mg/kg in citrate buffer, pH 4.5, Sigma-Aldrich) in 8 week-old C57/BL6 male mice as described in the previous report [9]. This murine model is representative of Type 1 diabetes mellitus. The control mice received citrate buffer alone. STZ-induced diabetic mice were euthanized under isoflurane anesthesia at 4, 8, 12 and 24 weeks after the completion of STZ injection. Control mice were euthanized by same method at 12 weeks after the completion of citrate buffer without STZ. Each group was comprised of eight mice and was evaluated at 4, 8, 12 and 24 weeks following STZ treatment.

Male db/db mice (BKS.Cg*-m+/+Leprdb*/BomTac, Samtakobiokorea, South Korea) were used as a model of Type 2 diabetes mellitus. Control and db/m (heterozygote) male mice were obtained at the same time from the same company. The three study groups were euthanized under isoflurane anesthesia at 10 and 16 weeks of age. All kidneys were perfused with 4 °C phosphate-buffered saline (PBS) and then excised. Half of the kidney was fixed in 10% neutralized formalin for immunohistochemistry and the remaining portions were used for quantitative polymerase chain reaction (qPCR) analyses as detailed below. Table 1 summarized the characteristics of both diabetic mice model including body weight, kidney weight and albuminuria.

**Table 1:** Charateristics of diabetic mice models

DM	variables		control (12wks)	STZ 4wks	STZ 8wks	STZ 12wks	STZ 24wks	*p*-value
Type 1	BW (gm)	Initial	23(23–23.8)	23.5(23–24)	23.5(22.8–24)	23(23–23)	23(23–24)	0.684
		Last	31(29.2–32.8)	27(26.3–27.3)	26(25–28.5)	26(25–27.8)	29(27–32)	0.001
	KW/BW (mg/mg)		0.005 (0.005–0.006)	0.008 (0.007–0.010)	0.008 (0.008–0.008)	0.008 (0.007–0.008)	0.009 (0.008–0.011)	< 0.001
	ACR (μg/mg)		48.0 (31.6–58.9)			90.6 (78.5–97.5)	70.3 (39.0–101.5)	0.002
DM	variables		control	db/db	db/db			*p*-value
			(db/m 10wks)	(10wks)	(16wks)			
Type 2	BW	Initial	24(24–26)	45(45–46)	45.5(43.5–47.5)			0.043
		Last	25(25–28)	48(45–49)	55(50.5–58.8)			0.001
	KW/BW (mg/mg)		0.006 (0.005–0.008)	0.004 (0.004–0.004)	0.004 (0.004–0.005)			0.045
	ACR (μg/mg)		77.5 (75.9–84.2)	1000 (702.5–2274.0)	1333 (975.3–3755.0)			0.05

Abbreviations: *DM* diabetes mellitus; *STZ* streptozotocin; *BW* body weight; *KW/BW* kidney weight/body weight; *ACR* albumin creatinine ratio

### Quantitative polymerase chain reaction (PCR) analysis

The FL-MMP-2 and NTT-MMP-2 mRNA expression levels were measured by quantitative RT-PCR (qPCR). The procedures were described in the previous report in detail [9]. *β*-actin as the housekeeping internal control was quantified in parallel with the target genes and all products were verified by melting curve analysis (95 °C 15 s, 60 °C 15 s, 95 °C 15 s). Normalization and the fold-changes for each of the genes were calculated using the 2^-ΔΔCT^ method. The primers used for qPCR are summarized in Table 2.

**Table 2:** Quantitative polymerase chain reaction primer sequences

Genes	Forward (5’➔3′)	Reverse (5’➔3′)
Full length MMP-2 (mouse)	GACCTCTGCGGGTTCTCTGC	TTGCAACTCTCCTTGGGGCAGC
N-terminal truncated MMP-2 (mouse)	GTGAATCACCCCACTGGTGGGTG	TTGCAACTCTCCTTGGGGCAGC
β-actin (mouse control)	CTCTCTTCCAGCCTTCCTTCC	CTCCTTCTGCATCCTGTCAGC

### Microscopic analysis/immunohistochemistry

The tissues were fixed in 10% formalin immediately after collection. Subsequently, the tissues were paraffin-processed and embedded. The periodic acid-Schiff (PAS) stain and Masson’s trichrome (MT) stain were performed to analyze any diabetic tissue injury and fibrosis. In addition, immunohistochemistry was performed on formalin-fixed 3 μm thick paraffin embedded sections. All of the procedures were conducted in the same way as the previous paper [9]. An isoform-specific antibody for NTT-MMP-2 targets the S1’ substrate binding loop as reported in detail [5] and antibody to FL-MMP-2 was purchased from Abcam (ab3158, Abcam, Cambridge, UK). Simultaneouly, immunostaining using negative control omitting the primary antibody was conducted to ensure the specificity of the immunodetection. The semi-quantitative staining grade was measured as follows: grade 0, negative staining; grade 1, weak patchy staining; grade 2, weak diffuse or dense patchy staining; and grade 3, dense diffuse staining. For the assessement of oxidative stress in the diabetic kidneys, immunostaining for the marker of oxidative stress, nitrotyrosine was performed with mouse monoclonal antibody [EM-30] to nitrotyrosine (ab125106, Abcam, Cambridge, UK). We conducted correlation analysis between NTT-MMP-2 transcript abundance and nitrotyrosin IHC staining score. For two isoforms of MMP-2 transcript abundance, we used the 2^-ΔCT^ method of analysis. Nitrotyrosin IHC scoring was performed as follows: 0, negative staining; 1, weak patch staining; 2, weak diffuse staining or dense patch staining; 3, dense diffuse staining.

### Statistical analysis

All statistical analyses were performed using GraphPad Prism 6.0 (GraphPad Software). The Kruskal-Wallis test with Dunn’s multiple comparison test or Mann Whitney U test were used to compare the experimental groups where appropriate. The spearman correlation analysis was conducted to prove the correlation between nitrotyrosine score and transcripts of both isoforms of MMP-2. A *p*-value of less than 0.05 was considered significant. The results are presented as the median (interquartile range) for all experiments.

## Results

### Streptozotocin model of type 1diabetes mellitus

**Fig. 1:**
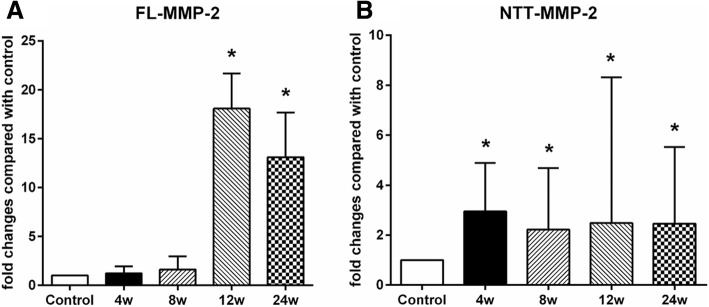
Quantitative PCR measurement FL-MMP-2 (**a**) and NTT-MMP-2 (**b**) transcript levels in the STZ model of Type 1 diabetes mellitus. qPCR performed on transcripts isolated from whole kidneys of controls and following 4, 8, 12, and 24 weeks after STZ injection. (*N* = 8 for each group; * *p* < 0.05)

Quantitative PCR showed that the FL-MMP-2 and NTT-MMP-2 transcripts were increased as compared to the non-diabetic control mice as function of the duration of diabetes (Fig. 1-a, b). In the case of FL-MMP-2, transcript abundance measured by quantitative PCR was significantly increased by 18.2 (14.0–21.7) fold and 13.1 (1.6–17.7) fold at 12 weeks and 24 weeks, respectively (*p* < 0.01). In the case of NTT-MMP-2, the transcripts were significantly increased 3.0 (2.3–4.9) fold at 4 weeks after the last STZ injection. NTT-MMP-2 transcript abundance was increased 2.5 (1.5–8.3) fold and 2.5 (2.1–5.5) fold at 12 and 24 weeks, respectively, *p* = 0.017). These experiments indicate that expression of the FL-MMP-2 and NTT-MMP-2 isoform transcripts have distinct temporal patterns in the STZ model of Type 1 diabetes mellitus, with NTT-MMP-2 isoform expression occurring significantly earlier than that of FL-MMP-2.

**Fig. 2:**
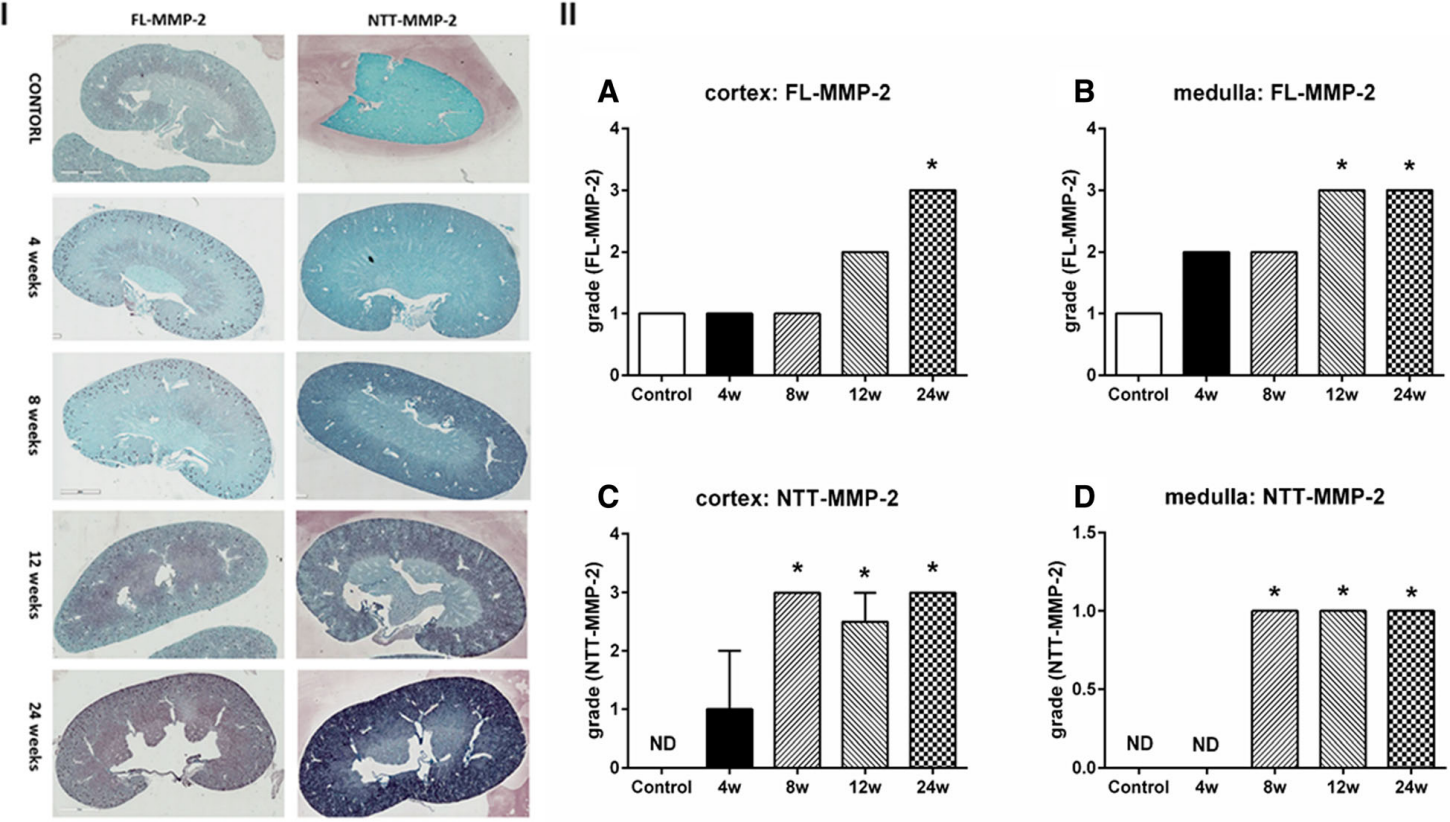
Immunohistochemical staining of kidneys for FL-MMP-2 and NTT-MMP-2 in the STZ model of Type 1 diabetes mellitus. MMP-2 isoform specific IHC was performed as detailed in Methods. Panel **I**: IHC staining for FL-MMP-2 and NTT-MMP-2 for controls and at 4, 8, 12 and 24 weeks following STZ treatment. There is low basal staining for the FL-MMP-2 isoform in the cortex of control kidneys, while NTT-MMP-2 is not detected. IHC staining for both isoforms progressively increases as a function of time and staining is most prominent in the renal cortex. Panel **II**: Semi-quantitative scoring of IHC staining. (*N* = 8 for each group; * *p* < 0.05; X10))

Figure 2 summarizes the results for IHC staining of renal cross sections for the FL-MMP-2 and NTT-MMP-2 isoforms in the STZ model. As shown in Fig. 2, panel I, there is low, but detectable expression of the FL-MMP-2 isoform in the renal cortex of control kidneys. In parallel with the qPCR studies of FL-MMP-2 transcript abundance, there no significant increase in IHC staining at 4 and 8 weeks. Prominent IHC staining for the FL-MMP-2 isoform was evident at 12 and 24 weeks in both the cortex and medulla. At both time points, cortical staining was comparable with medullary staining. Panel II, A and B, summarize semi-quantitative scoring of IHC staining for the FL-MMP-2 isoform.

In contrast to the FL-MMP-2 isoform, NTT-MMP-2 was not detectable by IHC in the either the cortex or medulla of control kidneys. At 4 weeks following STZ injection, NTT-MMP-2 signal was present primarily in the renal cortex and the intensity increased at 8 weeks and was primarily limited to the cortex. Panel II, C and D summarize semi-quantitative IHC scoring for the NTT-MMP-2 isoform.

### Db/db model of type 2 diabetes mellitus

**Fig. 3:**
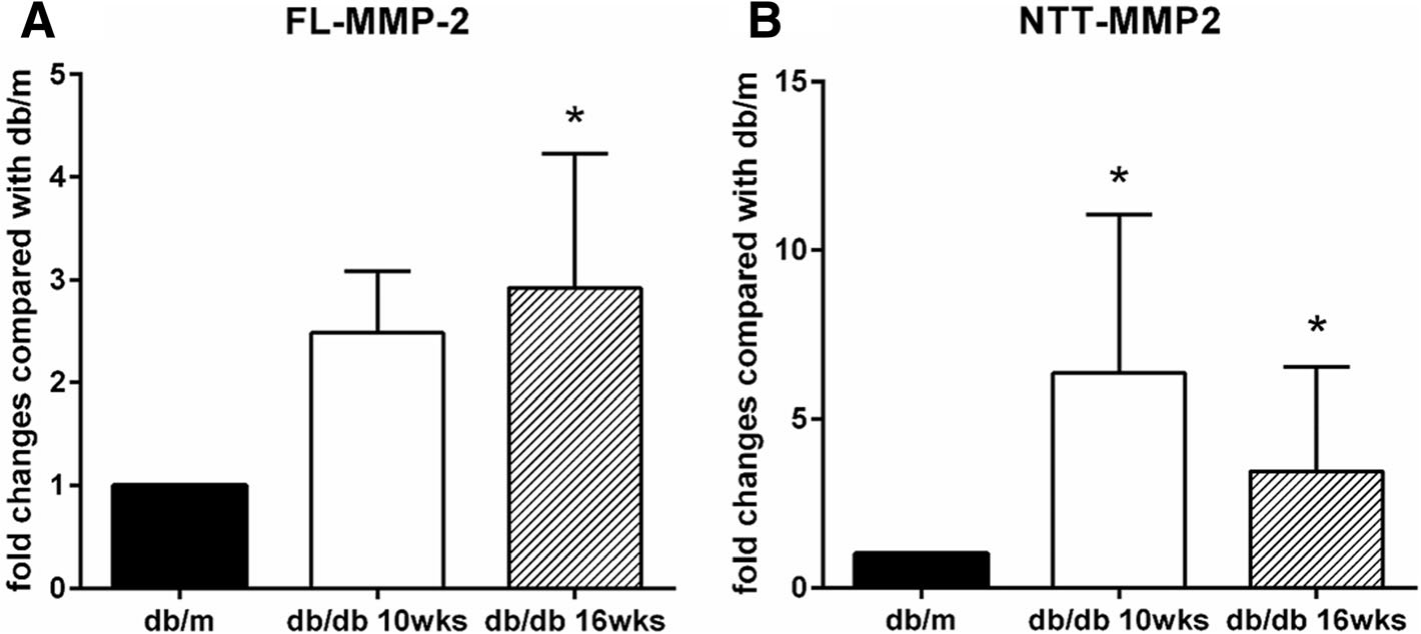
Quantitative PCR measurement of FL-MMP-2 (**a**) and NTT-MMP-2 (**b**) transcript levels in the db/db model of Type 2 diabetes mellitus. qPCR performed on transcripts isolated from while kidneys of db/m controls and from db/db mice at 10 and 16 weeks of age. (N = 8 for each group; * *p* < 0.05)

**Fig. 4:**
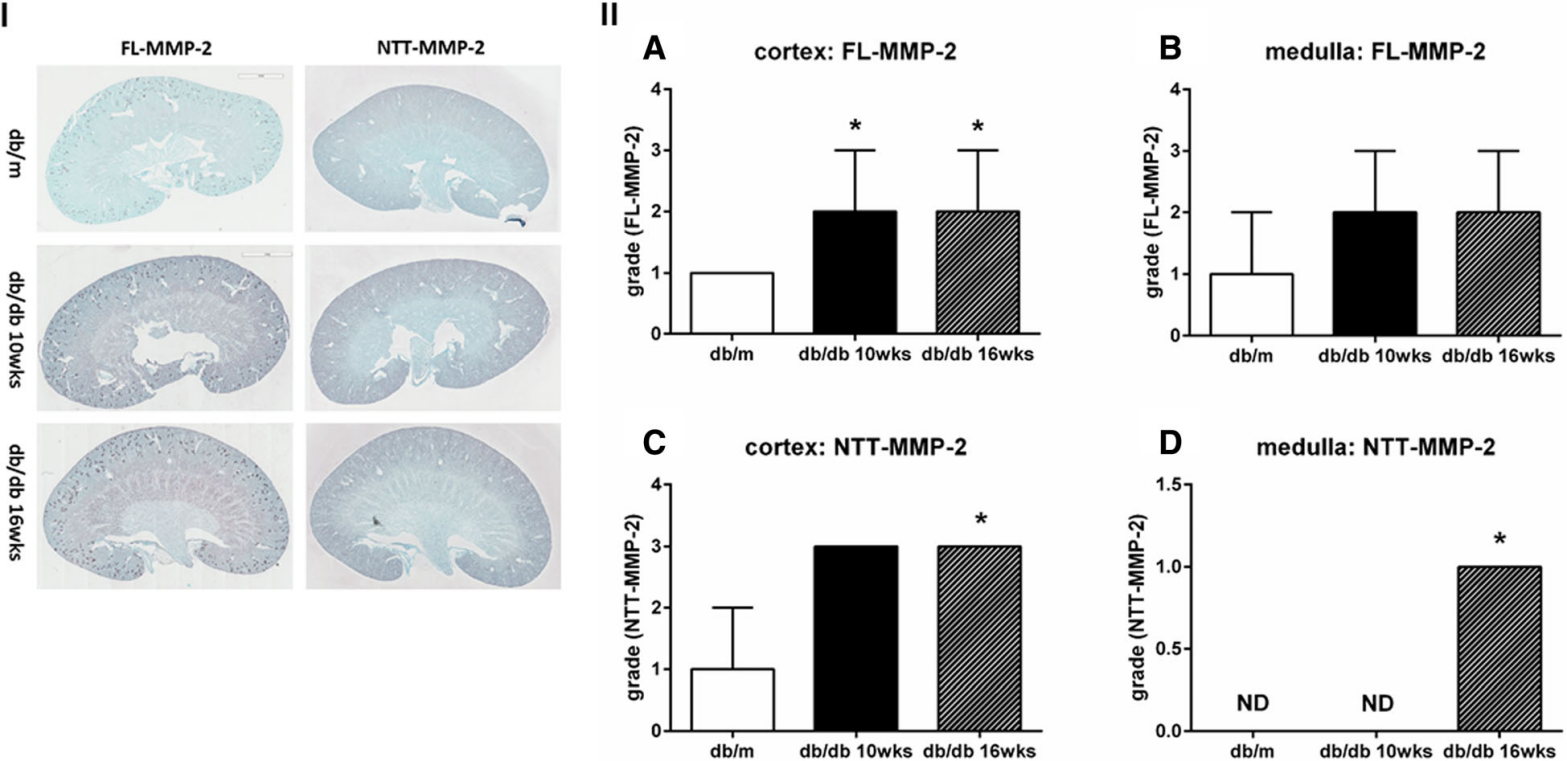
Immunohistochemical staining of kidneys from FL-MMP-2 and NTT-MMP-2 in the db/db model of Type 2 diabetes mellitus. Panel **I**. There is a low basal expression of FL-MMP-2 and NTT-MMP-2 in the cortex of control db/m kidneys. Staining for both isoforms increases in the db/db kidneys at 10 and 16 weeks. Panel **lI**. Semi-quantitative scoring of IHC staining. (N = 8 for each group; * *p* < 0.05; X10)

Quantitative PCR results for expression of the two MMP-2 isoforms in the kidney using the db/db model of Type 2 diabetes mellitus are summarized in Fig. 3. Expression of the FL-MMP-2 transcript in kidneys was not significantly increased until 10 weeks of age, and was only significantly increased at 16 weeks of age by approximately three-fold as compared to controls. In contrast, NTT-MMP-2 transcript abundance was increased nearly 6-fold by 10 weeks of age. Figure 4 summarizes the results for IHC staining for the MMP-2 isoforms in the db/db model kidneys. The FL-MMP-2 isoform was detected in both the cortex and medulla at 10 and 16 weeks of age, in each case expression of the FL-MMP-2 in the cortex was comparable with the medulla. Panel II, A and B summarize the semi-quantitative IHC staining for the FL-MMP-2 isoform. The NTT-MMP-2 isoform was detected mainly in the renal cortex, but not prominent in the medulla of db/db kidneys. Panel II, C and D summarize the semi-quantitative IHC staining for the NTT-MMP-2 isoform.

**Fig. 5:**
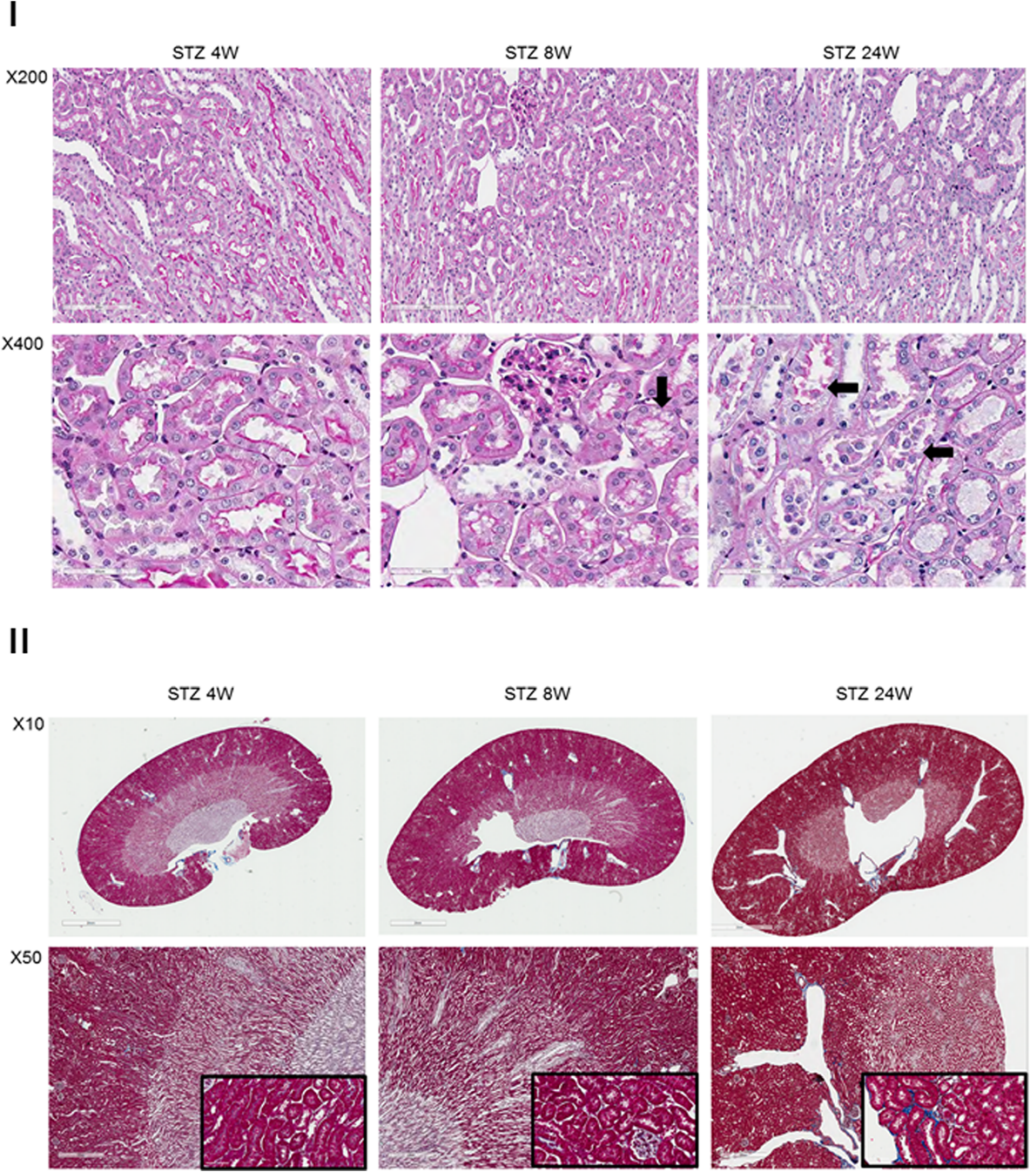
PAS and Masson trichrome staining of kidneys from the STZ model of Type 1 diabetes mellitus. Panel **I**: PAS staining of renal cortex at 4, 8 and 24 weeks after STZ treatment. PAS staining is normal in the 4 week STZ group, while vacuolization of tubular epithelial cells is present at 8 weeks (arrow). By 24 weeks there is widespread tubular epithelial cell necrosis with cellular debris in the tubular lumens (arrows). Panel **II**: Masson trichrome staining of kidneys at 4, 8 and 24 weeks following STZ treatment. There is no detectable fibrosis at 4 and 8 weeks, while there are small foci of fibrosis (blue staining) in the cortex of kidneys at 24 weeks (inset X400)

We performed PAS and Masson trichrome staining on the kidneys from STZ-treated and db/db mice to determine if a correlation exists between the timing of MMP-2 isoform expression and tubular injury and fibrosis. Representative PAS-stained sections for the STZ-treated mice are shown in Fig. 5, Panel I. Masson trichrome-stained sections are shown in Fig. 5, Panel II. At four weeks following STZ treatment the PAS and Masson trichrome sections are histologically normal. At 8 weeks following STZ treatment, a time at which NTT-MMP-2, but not FL-MMP-2 was upregulated (Figs. 1, 2) there was evidence for cytoplasmic vacuolization of the proximal tubule epithelial cells; however, there was no evidence for fibrosis as determined by Masson trichrome staining. By 24 weeks following STZ treatment when both the FL-MMP-2 and NTT-MMP-2 isoforms are upregulated, there was evidence for widespread proximal tubular epithelial cell necrosis with tubular lumens filled with necrotic cells in PAS-stained renal sections. In contrast to the widespread tubular epithelial cell necrosis at 24 weeks, there were only rare small foci of fibrosis.

**Fig. 6:**
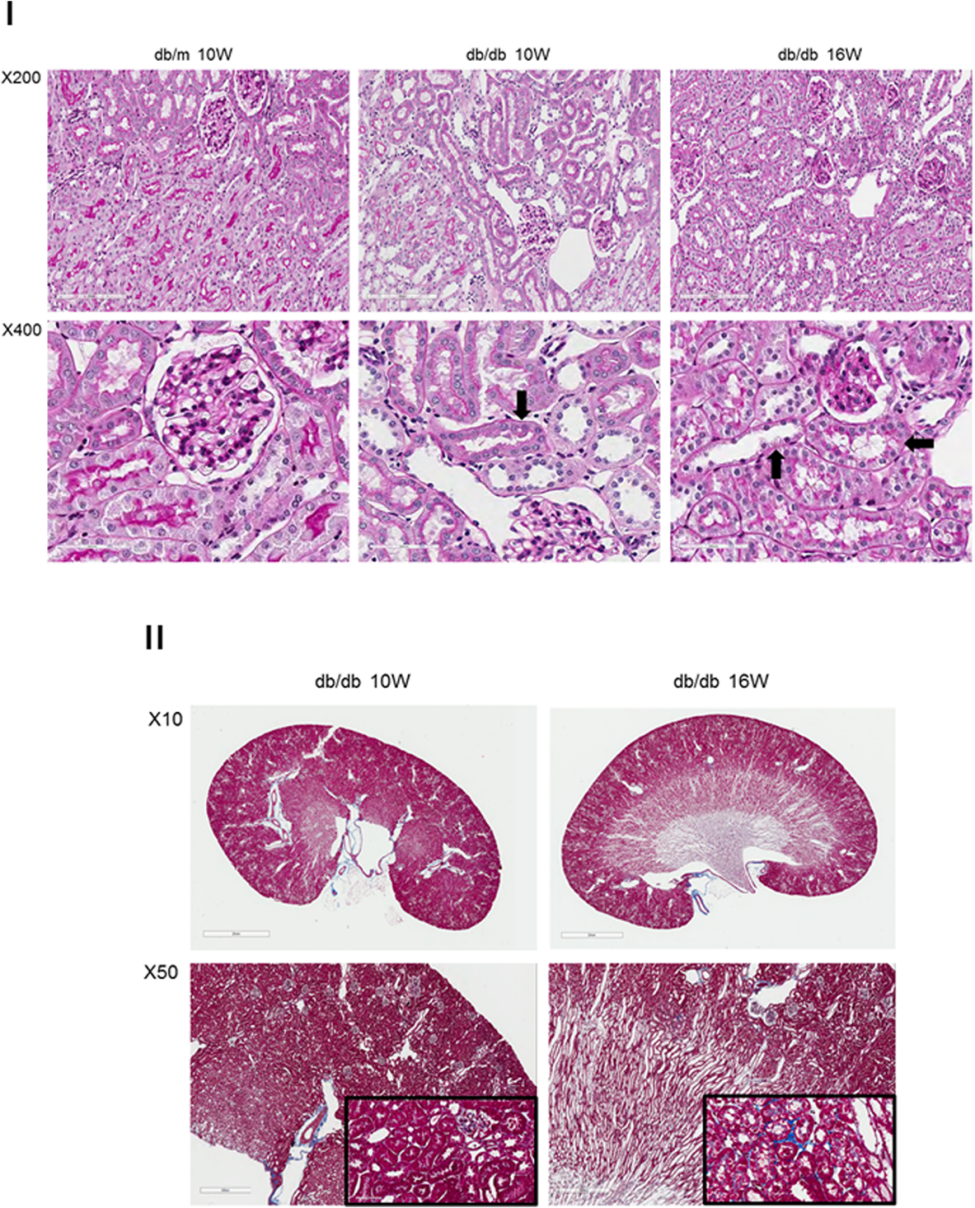
PAS and Masson trichrome staining of kidneys from the db/db model of Type 2 diabetes mellitus. Panel **I**: PAS staining of control db/m kidneys is normal. At 10 weeks of age kidneys of db/db mice show occasional tubular epithelial cell vacuolization (arrow), which is much more extensive at 16 weeks of age (arrows). Panel **II**. Masson trichrome staining of control db/db kidneys at 10 and 16 weeks of age. There are rare, scattered foci of fibrosis (blue) in the deep cortex at 16 weeks, but not at 10 weeks of age (inset X400)

We performed similar analyses with PAS and Masson trichrome staining on the db/db mice at 10 and 16 weeks of age. Representative results of these studies are shown in Fig. 6, Panels I and II. Proximal tubular epithelial cell vacuolization was present in the 10 week old db/db kidneys and by 16 weeks there was evidence for tubular epithelial cell necrosis with tubular dilation. Masson trichrome staining revealed only occasional patchy foci of fibrosis in the 16 week old db/db mice, which were not present in the 10 week old db/db mice.

**Fig. 7:**
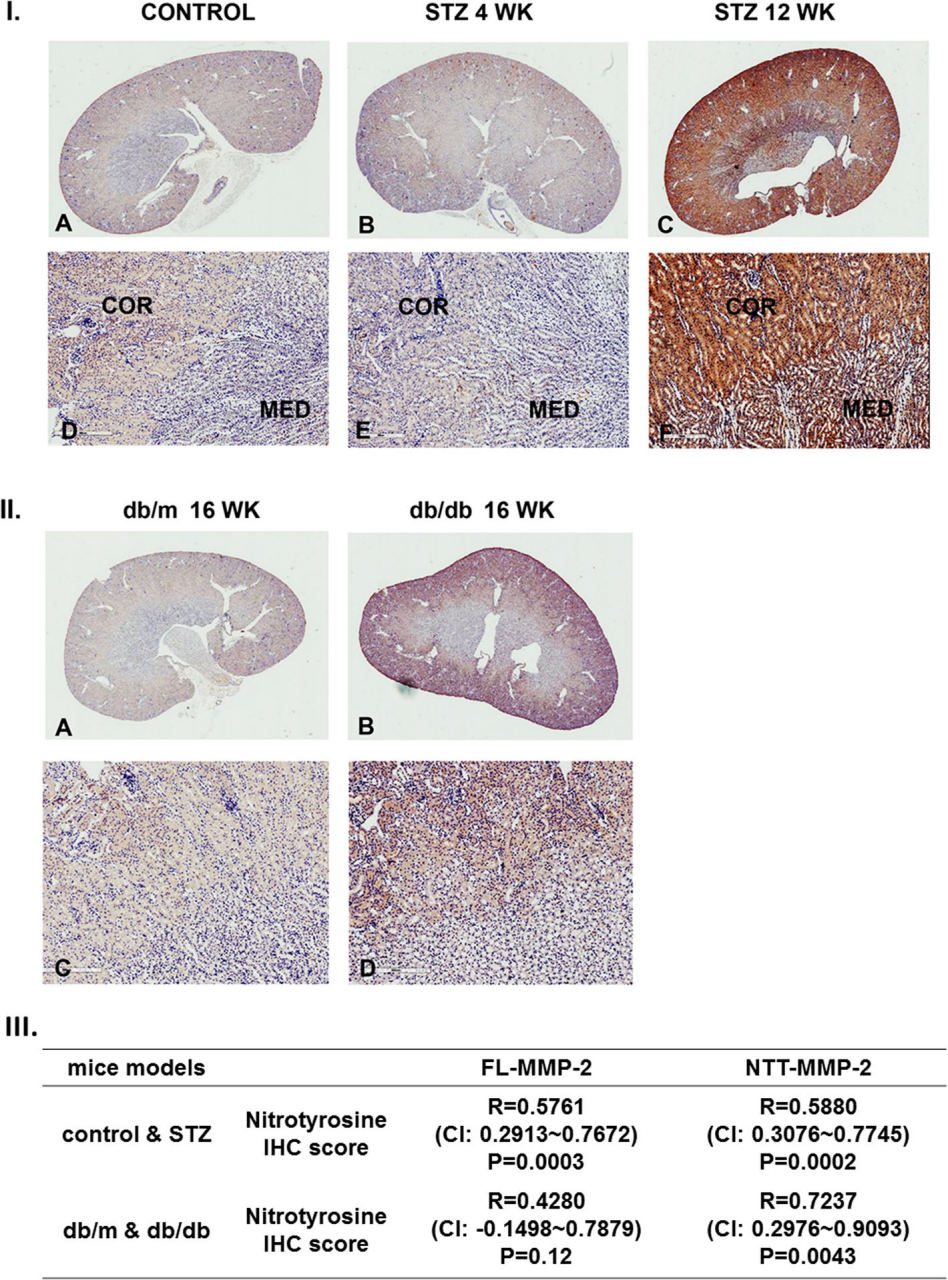
Nitrotyrosine IHC of kidneys from STZ-treated and db/db mice. Panel **I**: Nitrotyrosine IHC from control kidneys (*a*, *d*) and at 4 weeks (b, *e*)and 12 weeks (*c*, *e*) following STZ treatment. IHC staining for nitrotyrosine is absent in the controls but is detectable at 4 weeks and further increased at 12 weeks. Nitrotyrosine IHC staining is most prominent within the cortex as compared to the medulla. (*a*-*c* X10; D-F X100). Panel **II**: Nitrotyrosine IHC of kidneys from control db/m (*a*, *c*) and with 16 week old db/db kidneys (b, d). Nitrotyrosine IHC staining is concentrated in the cortex of the 16 week old db/db kidneys and is not detected in the age-matched db/m controls. (*a*, *b* X 10; C,D X100). Panel **III**: Nitrotyrosin IHC score is correlated with two isoforms of MMP-2 in STZ and control kidneys. In db/m and db/db kidneys, NTT-MMP-2 is only correlated with nitrotyrosine ICH score. (R, correlation coefficient; CI, 95% confidence interval; ICH, immunohistochemistry)

We have recently reported that transgenic expression of the NTT-MMP-2 isoform in renal proximal tubular epithelial cells induces oxidative stress mediated by initiation of the mitochondrial permeability transition [5]. We used IHC staining for nitrotyrosine as a marker of oxidative stress and representative results of these studies are presented in Fig. 7. As compared to controls, there was no increase in nitrotyrosine IHC staining at 4 weeks following STZ treatment; however, there was prominent staining for nitrotyrosine at 12 weeks following STZ treatment. Notably, nitrotyrosine staining was considerably stronger in the cortex as opposed to the medulla. Nitrotyrosine staining was not detectable in the kidneys of 10 weeks old db/m kidneys, but was readily detected in kidneys of 16 week old db/db mice (Fig. 7-I, II). As with the STZ-treated mice, nitrotyrosine staining was much more abundant in the cortex as compared to the medulla. The temporal patterns and expression levels of nitrotyrosine expression, a marker of oxidative stress, correlated with qPCR determination of MMP-2 isoform expression (Fig. 7-III).

## Discussion

The principal findings of this study are that two discrete MMP-2 isoforms are induced in kidneys of murine models representative of Type 1 and Type 2 diabetes. In particular, the expression patterns of FL-MMP-2 and NTT-MMP-2 differed according to the anatomical location and the length of time with hyperglycemia. In the context of the time of a diabetic milieu, NTT-MMP-2 was more significantly induced earlier in both murine diabetic models as compared to FL-MMP-2. In the context of the anatomical location, although the two isoforms of MMP-2 were expressed gradually in the renal cortical tubular segment according to the time of the two different diabetic milieu, the expression FL-MMP-2 in the outer medulla was more prominent as compared to NTT-MMP-2. Interestingly, NTT-MMP-2 was not expressed in whole kidney of normoglycemic control mice, while low cortical levels of FL-MMP-2 were detected by IHC in normoglycemic kidneys. In addition, although IHC staining for NTT-MMP-2 revealed low, detectable levels in the cortex of the db/m mice, the degree was faint and its expression was not evident in the outer medulla of db/m mice. In the later stage of the STZ induced diabetic model, NTT-MMP-2 was upregulated strongly in the cortex compared to the medulla. In the db/db mice, the two isoforms of MMP-2 were more significantly upregulated in cortex as compared with medulla. Based on these findings and previous data, the abundance of FL-MMP-2 and NTT-MMP-2 in the two diabetic nephropathy models may be dependent on the degree of hyperglycemia and the degree of associated oxidative stress. Importantly, we showed that the upregulation of NTT-MMP-2 preceded evidence for the tubular injury pattern as previously reported in our transgenic model expressing NTT-MMP-2 in the renal proximal tubule [6].

To date, studies have provided conflcting data about the roles of matrix metalloproteinases in both experimental and clinical diabetic nephropathy [10]. MMP-2 can be expressed in the whole segments anatomically in the animal kidney, even though those kidneys were obtained from the rabbit, rats, and monkeys [11]. The present study is valuable because it defines the anatomical location (cortex vs. medulla) and the time of induction of MMP-2 isoform induction by hyperglycemia in both type 1 and type 2 murine diabetic models.

The NTT-MMP-2 isoform was initially characterized in isolated mitochondria from a murine model of systolic heart failure and accelerated atherogenesis and was also detected in the cardiac mitochondrial fraction of aged mice [8]. Subsequently, the NTT-MMP-2 isoform expression significantly correlated with tubular epithelial cell necrosis in human renal transplant delayed graft function [7]. Hyperglycemia- induced oxidative stress is considered as a major driver in diabetic nephropathy [12–14]. We have demonstrated that oxidative stress induces NTT-MMP-2 synthesis via activation of an alternate promoter located in the first intron of the MMP-2 gene [8]. Further, NTT-MMP-2, per se, induces further oxidative stress via induction of the mitochondrial permeability transition, with resultant tubular epithelial cell regulated necrosis [5]. The present study shows that the nitrotyrosine expression in the kidney is associated with the two isoforms of MMP-2, especially NTT-MMP-2. In addition, the hyperglycemia and mitochondrial pathway of regulated necrosis can be related to the increased susceptibility of rodent models and human with diabetes to ischemia-reperfusion injury [15].

## Conclusions

Two isoforms of MMP-2 are consistently highly inducible in murine models representative of type 1 and type 2 diabetes and gradually become abundant over time of the diabetic milieu. As the expression patterns were not the same in the two isoforms of MMP-2, it is possible that these isoforms have a respective role in diabetic renal injury.

## Abbreviations

DM: Diabetes mellitus
ESRD: End-stage renal disease
FL-MMP-2: The full length matrix metalloproteinase-2
MMP-2: Matrix metalloproteinase-2
MT: Masson’s trichrome
NTT-MMP-2: N-terminal truncated isoform
PAS: Periodic acid-Schiff
qPCR: Quantitative polymerase chain reaction
STZ: Streptozotocin
